# Hospitalizations due to Alzheimer’s disease in Brazil during the COVID-19 pandemic: an update on frequency, mortality, and costs

**DOI:** 10.1590/1980-5764-DN-2025-0322

**Published:** 2026-01-09

**Authors:** Pedro Carrión Carvalho, Giovana Busnardo Voltolini, Amanda Goedert, Vitória Kovari Carmona Chiaratti, Eduardo Holsbach Cantarelli, Júlia Costa Francisco, Tanize Bechorner Almeida, Ismael Paulo Burigo

**Affiliations:** 1 Centro Universitário de Brusque, Faculdade de Medicina, Brusque SC, Brazil.; 2 Universidade Federal de Santa Maria, Santa Maria RS, Brazil.

**Keywords:** Alzheimer Disease, Hospitalization, Length of Stay, Health Care Costs, Brazil, Doença de Alzheimer, Hospitalização, Tempo de Internação, Custos de Cuidados de Saúde, Brasil

## Abstract

Hospitalizations related to Alzheimer’s disease (AD) impose a growing burden on health systems, but recent, nationally representative estimates for Brazil are limited.

**Objective:**

To describe the epidemiological profile of hospital admissions due to AD in Brazil from 2018 to 2024.

**Methods:**

Ecological time-series study using the Hospital Information System of the Brazilian Unified Health System (SIH/SUS), accessed via the Department of Informatics of the SUS (DATASUS). We included all regions and states from January 2018 to December 2024. Admissions were identified by the International Classification of Diseases, 10^th^ Revision (ICD-10) codes G30.0–G30.9 and F00.0–F00.9. Variables comprised sex, age group, race/color, admission type (urgent/elective), in-hospital mortality, length of stay, and hospital costs. Temporal trends were evaluated with linear regression.

**Results:**

From 2018 to 2024, 11,212 AD-related hospitalizations were recorded; 79.4% were urgent. The Southeast had the highest absolute number (47.8%), followed by the South (25.1%), Northeast (17.2%), Midwest (6.5%), and North (3.4%). Females accounted for 65% of admissions and 64.7% of in-hospital deaths. Older adults, especially those ≥80 years, represented most hospitalizations (59.3%) and deaths (69.7%). Total hospital expenditures exceeded R$ 14 million, with the Southeast concentrating >60% of national costs. No significant linear trend was detected in annual rates.

**Conclusion:**

Urgent admissions comprised the majority of AD hospitalizations nationwide, with the Southeast presenting the highest numbers. The predominance of older female patients and high in-hospital mortality underscore the need for targeted clinical and public health strategies. Rising expenditures reinforce investment in health infrastructure and long-term dementia-care policies in Brazil.

## RESUMO.

As internações por doença de Alzheimer (DA) impõem carga crescente aos sistemas de saúde, mas estimativas nacionais recentes e representativas para o Brasil são limitadas.

**Objetivo:**

Descrever o perfil epidemiológico das internações por DA no Brasil entre 2018 e 2024.

**Métodos:**

Estudo ecológico de séries temporais com dados do Sistema de Informações Hospitalares do SUS (SIH/SUS), acessados pelo Departamento de Informática do SUS (DATASUS). Incluíram-se todas as regiões e estados, de janeiro/2018 a dezembro/2024. As internações foram identificadas pelos códigos da 10ª Revisão da Classificação Internacional de Doenças (CID-10) G30.0–G30.9 e F00.0–F00.9. As variáveis incluíram sexo, faixa etária, raça/cor, tipo de internação (urgente/eletiva), mortalidade intra-hospitalar, tempo de internação e custos hospitalares. Tendências temporais foram avaliadas por regressão linear.

**Resultados:**

No período, foram registradas 11.212 internações por DA; 79,4% foram urgentes. O sudeste apresentou o maior número absoluto (47,8%), seguido pelo sul (25,1%), nordeste (17,2%), centro-oeste (6,5%) e norte (3,4%). As mulheres corresponderam a 65% das internações e 64,7% dos óbitos intra-hospitalares. Idosos, especialmente ≥80 anos, concentraram a maioria das internações (59,3%) e dos óbitos (69,7%). Os gastos hospitalares totalizaram >R$ 14 milhões, com o sudeste concentrando >60% do custo nacional. Não houve tendência linear significativa nas taxas anuais.

**Conclusão:**

As internações urgentes predominaram no país, com o sudeste apresentando os maiores números. A predominância de idosas e a elevada mortalidade reforçam a necessidade de estratégias dirigidas em manejo clínico e saúde pública. O aumento dos gastos sustenta investimentos em infraestrutura e em políticas de cuidado de longa duração para demência no Brasil.

## INTRODUCTION

 Dementia is a term used to describe a significant decline in cognitive function that interferes with a person’s daily activities^1^. Alzheimer’s disease (AD) is the most common and irreversible form of dementia, accounting for approximately 60–70% of all cases worldwide^2,3^. According to the Global Burden of Disease Study 2019, an estimated 57 million people are currently living with dementia globally—a number expected to triple by 2050^4^. In Brazil, the 2024 National Dementia Report by the Ministry of Health estimated a prevalence of 5.2% among individuals aged 60 and over, with AD being the most frequent subtype^5^. Recent findings by Aliberti et al.^6^ further underscore the growing burden of dementia in the Brazilian population, particularly in the context of population aging and regional disparities in access to long-term care^7^. 

 The histopathological features found in individuals with AD include the presence of extracellular senile plaques composed of filamentous aggregates of beta-amyloid protein, accumulation of abnormal tau protein filaments with the consequent formation of neurofibrillary tangles, neuronal and synaptic loss, glial activation, and inflammation^5,7^. There are two main hypotheses to explain its etiology: the cholinergic hypothesis, which proposes that the early death of cholinergic neurons impairs cognitive processes, and the amyloid hypothesis, which posits that neuronal toxicity is caused by amyloid deposits—the most widely accepted theory to date^1^. 

 AD is a complex disorder responsible for nearly three-quarters of all dementia cases^4,5,7^. It presents different progressive stages, with symptoms varying depending on the stage of the disease^4,5,7^. The early stages may be asymptomatic and show no apparent neurological impairment, which can last for years. As the neurodegenerative disorder progresses, it may evolve to more advanced and critical stages, characterized by short-term memory loss, impacting daily activities and personal relationships^4,7^. Over time, symptoms worsen, ultimately leading to loss of independence^4,7^. 

 The number of dementia cases has been increasing in line with Brazil’s demographic transition, making this a highly relevant topic and a growing public health concern. Globally, the number of people affected by dementia is estimated to have increased by 117% between 1990 and 2016, largely due to population aging^8^. As for treatment, no effective drugs have yet been discovered to reverse or cure the disease. Therefore, current therapies are solely symptomatic and palliative. These patients often require multidisciplinary care due to a decline in quality of life and loss of autonomy, which increases morbidity and the likelihood of hospitalization^9^. Moreover, hospital morbidity in AD is primarily due to the severity of cognitive deterioration, which leads most patients in the advanced stages of the disease to prolonged hospital stays and in-hospital death, generally from secondary causes^10,11^. 

 Hospitalizations can be classified as urgent or elective. Urgent hospitalizations are unplanned and immediate, resulting from an acute episode or sudden complication. These situations involve patients in critical condition requiring immediate stabilization^12,13^. This type of care aims to resolve a crisis or achieve rapid stabilization, with the most common examples being falls, extreme agitation, delirium, and severe infections^13-15^. Elective hospitalization, in contrast, is planned in advance, following a medical evaluation and requiring monitoring, treatment adjustment, diagnostic testing, or rehabilitation in patients who are clinically stable^13-17^. 

 Given the substantial public health burden, high prevalence, functional impairment, and the accelerating aging of the population, it is essential to characterize the epidemiological profile of AD-related hospitalizations in Brazil. This includes identifying the most affected regions and demographic groups, as well as analyzing trends in hospital morbidity, mortality, and the distribution of urgent versus elective admissions. This study aims to provide a national overview of AD-related hospitalizations and discuss its implications for healthcare planning and resource allocation. 

## METHODS

### Search protocol

 This is an analytical, observational, longitudinal, and retrospective ecological study. Data were collected from the Hospital Information System of the Brazilian Unified Health System (SIH/SUS), provided by the Department of Informatics of the SUS (DATASUS), through the official TABNET platform^18^. As all data are publicly available and anonymized, submission to a Research Ethics Committee was not required, in accordance with national guidelines for secondary data research. 

 The search was conducted between March 3 and March 10, 2025. The epidemiological and morbidity database "Hospital Morbidity of SUS (SIH/SUS)" was accessed, selecting the option "General by place of hospitalization from 2008," with the geographic scope set to "Brazil by Region and State." The filters used included regions (South, Southeast, Midwest, Northeast, and North); Chapter VI (Diseases of the Nervous System) of the International Classification of Diseases, 10^th^ Revision (ICD-10); and the ICD-10 Morbidity List codes G30.0 to G30.9 (Alzheimer’s disease) and F00.0 to F00.9 (Dementia in Alzheimer’s disease)^19^. These codes were included to increase diagnostic sensitivity, as both refer to the same underlying clinical condition from different etiological perspectives (neurological and psychiatric). 

 Variables analyzed included the number of hospitalizations and deaths, segmented by year, sex, age group, type of admission (urgent or elective), average length of stay (in days), and hospital service costs. Four authors (P.C.C., A.G., G.B.V., and V.K.C.C.) independently extracted and validated the data based on predefined criteria. Microsoft Excel® and Apple Numbers® were used for data storage and descriptive analysis. 

### Statistical analysis

 Descriptive statistics were applied to summarize hospitalization patterns. 

 To assess temporal trends, we fitted ordinary least squares (OLS) models with year as a continuous predictor and the annual age-standardized hospitalization rate (per 100,000) as the primary outcome for 2018–2024. Model assumptions were examined by visual inspection of residuals (linearity, homoscedasticity, and normality of residuals); when heteroscedasticity was suggested, we reported robust (HC3) standard errors. As robustness checks, we modeled annual counts using generalized linear models for counts (Poisson and negative binomial) with a log link and an offset for the mid-year population; we also fitted analogous models for the hospital mortality rate and the average cost per hospitalization. All tests were two-sided with α=0.05. Analyses were performed in Python (SciPy) and spreadsheet software. 

 We summarized categorical variables as counts and percentages and continuous variables as means with standard deviations or medians with interquartile ranges, as appropriate. Differences in proportions (e.g., sex, urgent admissions, regional shares, in-hospital mortality) were assessed with two-proportion z-tests; absolute percentage-point differences were reported with 95% confidence intervals (CIs). Comparisons of rates across years or regions were expressed as rate ratios with exact (Poisson) 95% CIs. For length of stay (LOS), normality was assessed (Shapiro-Wilk and visual inspection); mean±standard deviation (SD) were compared with Welch’s t-test when approximately normal or median (interquartile range—IQR) with the Mann-Whitney/Kruskal-Wallis test otherwise. Cost comparisons used Welch’s variance analysis (ANOVA) or, when skewed, nonparametric tests; where relevant, generalized linear models with gamma distribution and log link were explored in sensitivity analyses. 

### Rates and age standardization

 Crude annual hospitalization rates for Alzheimer’s disease (AD) were calculated as the number of AD-related hospitalizations divided by the corresponding mid-year population and expressed per 100 thousand inhabitants. To enhance comparability over time, age-standardized rates (ASR) per 100 thousand were computed using the direct method across five age bands (60–64, 65–69, 70–74, 75–79, and ≥80 years). Age-specific rates were obtained by dividing yearly counts in each age band by the corresponding population denominators. Standard weights were derived from the Brazilian 2022 Census age distribution in these bands. Ninety-five percent CIs were presented for crude rates; ASR were displayed as point estimates. 

## RESULTS

### Hospitalizations by year (2018–2024)

**Figure 1:**
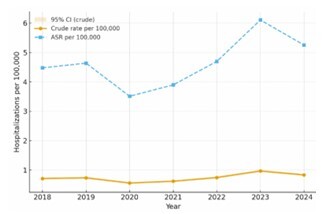
National Alzheimer’s disease hospitalization rates, Brazil, 2018–2024. The solid line shows crude rates per 100,000 inhabitants with 95% confidence intervals (shaded area). The dashed line shows agestandardized rates (ASR) per 100,000 using the direct method across five age bands (60–64, 65–69, 70–74, 75–79, and ≥80 years), with standard weights from the Brazilian 2022 Census.

 Between January 2018 and December 2024, a total of 11,211 hospitalizations due to AD were recorded in Brazil. The annual number of hospitalizations varied, with 1,524 cases in 2018, 1,584 in 2019, followed by a sharp drop to 1,199 in 2020 during the peak of the COVID-19 pandemic. In 2021, 1,353 hospitalizations were registered, followed by 1,627 in 2022. A notable increase was observed in 2023 with 2,125 cases, the highest recorded in the series, before declining slightly to 1,799 in 2024. National hospitalization rates for AD are presented both as crude rates per 100 thousand inhabitants and as ASR, with 95% CIs (Figure 1). In linear regression of annual age-standardized hospitalization rates (2018–2024), the slope was not statistically different from zero (p=0.1558), consistent with the visual pattern in Figure 1. 

### Type of admission and hospital stay

 Urgent admissions accounted for 8,907 cases (79.4%), while 2,305 admissions (20.6%) were elective. The difference in proportions between urgent and elective admissions was statistically significant; absolute percentage-point differences with 95% CIs and p-values are reported in Table 1. The average length of hospital stay differed markedly: urgent admissions averaged 25.9 days, while elective admissions averaged 51.8 days, indicating a more prolonged clinical approach in planned hospitalizations. These patterns were consistent across all five Brazilian macroregions (Table 1). 

**Table 1:** Hospitalizations due to Alzheimer’s disease in Brazil, 2018–2024 — regional distribution, admission type, and length of stay. Values are n and %, unless otherwise indicated. "Share (%)" denotes the proportion of national hospitalizations accounted for by each region. "Δ pp vs Southeast" indicates the absolute percentage-point difference in regional share compared with the Southeast, with 95% confidence intervals (CIs) and two-sided p-values from two-proportion z-tests. Urgent/elective percentages are within-region. Length of stay (LOS) is reported in days; summary metrics follow distributional assessment (mean±standard deviation—SD when approximately normal, otherwise median [interquartile range—IQR]).

Region	Hospitalizations	Share (%)	∆pp vs Southeast	95%CI (∆pp)	p-value	Urgent [n (%)]	Elective [n (%)]	LOS urgent (days)	LOS elective (days)
Northern	385	3.43	-44.35	-45.33 to -43.36	0.0	326 (84.7)	59 (15.3)	6.44	12.54
Northeastern	1,925	17.17	-30.61	-31.77–29.45	0.0	1.361 (70.7)	564 (29.3)	18.35	22.9
Southeast	5,357	37.78	0.0	-1.31–1.31	1.0	4.112 (76.8)	1,245 (23.2)	11.9	53.49
Southern	2,817	25.12	-22.65	-23.88 to -21.43	0.0	2.599 (92.3)	218 (7.7)	10.39	17.03
Midwest	728	6.49	41.29	-42.32 to -40.26	0.0	509 (69.9)	219 (30.1)	8.94	16.19
Total	11,212	100	-	-	-	8,907 (79.4)	2,305 (20.6)	-	37.97

### Hospitalizations by region

 The Southeast region had the highest numbers of AD-related hospitalizations, with 5,357 cases (47.8%), followed by the South (2,817; 25.1%), Northeast (1,925; 17.2%), Midwest (728; 6.5%), and North (385; 3.4%). Regional shares were compared with two-proportion tests, and pairwise absolute percentage-point differences are shown with 95%CIs and p-values in Table 1. 

### Demographic profile: sex, age, and race

 Women represented the majority of AD-related hospitalizations (7,299; 65%), whereas men accounted for 3,913 (35%). The observed difference in percentages was statistically tested, and the absolute percentage-point difference is presented with its 95%CI and p-value (Table 1). In terms of age, the group aged 80 years or older predominated with 6,654 hospitalizations (59.3%), followed by individuals aged 70–79 (3,151; 28.1%) and 60–69 (1,050; 9.4%). Younger adults under 60 represented only 3.2% of cases. Regarding race/color, hospitalizations were distributed as follows: White (5,700; 50.8%), Pardo (3,138; 28.0%), Black (510; 4.5%), Asian (156; 1.4%), Indigenous (4; <0.1%), and unknown (1,723; 15.4%) (Table 1). 

### Mortality

**Figure 2:**
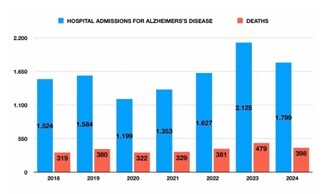
Annual counts of Alzheimer’s disease hospitalizations and inhospital deaths, Brazil, 2018–2024. Bars represent hospitalizations (left y-axis) and the line depicts in-hospital deaths (right y-axis). The lowest number of admissions and the highest in-hospital mortality rate occurred in 2020.

 A total of 2,608 hospital deaths due to AD were documented over the study period. Differences in in-hospital mortality proportions across sex, region and year were tested; absolute percentage-point differences with 95%CIs and p-values are provided in Table 1. Annual deaths were: 319 (2018), 380 (2019), 322 (2020), 329 (2021), 381 (2022), 479 (2023), and 398 (2024). The highest hospital mortality rate occurred in 2020 (26.9%), coinciding with the early phase of the COVID-19 pandemic. Overall, no statistically significant trend was observed in hospital mortality (p=0.8105). Of the total deaths, 2,064 (79.1%) occurred during urgent admissions, while 544 (20.9%) occurred during elective hospitalizations. Mortality was higher among women (1,687; 64.7%) and among patients aged 80 or older (1,819; 69.7%). By race, the deaths were distributed as follows: White (1,467), Pardo (646), Black (170), Asian (29), unknown (296), with no deaths recorded among Indigenous individuals (Figure 2). 

### Mortality by region and admission type

**Figure 3:**
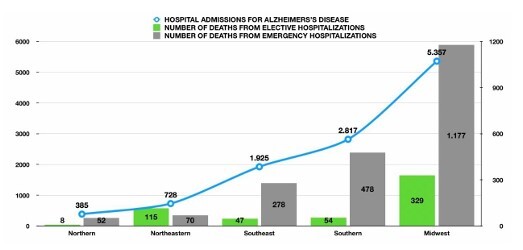
In-hospital deaths due to Alzheimer’s disease by admission type and region, Brazil, 2018–2024. Grouped bars show the number of deaths during urgent and elective hospitalizations within each region; markers indicate the proportion of deaths occurring during urgent admissions.

 The Southeast region also concentrated the largest number of in-hospital deaths (1,506; 57.8%), followed by the South (532; 20.4%), Northeast (325; 12.5%), Midwest (185; 7.1%), and North (60; 2.3%). Most deaths occurred during urgent hospitalizations in all regions. Of note, the Midwest had the highest proportion of deaths occurring during elective admissions (57.3%) (Table 1 and Figure 3). 

### Hospital costs

**Figure 4:**
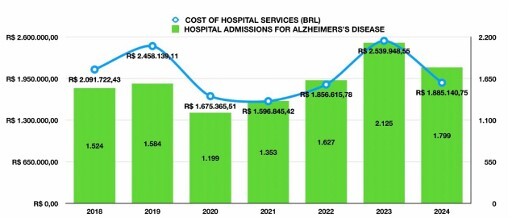
Average cost per Alzheimer’s disease hospitalization (BRL), Brazil, 2018–2024. Points and line show annual means; no significant linear trend was detected (p=0.1606). Values are nominal BRL (no inflation adjustment). Notes: Costs reflect approved Hospital Information System of the Brazilian Unified Health System (SIH/SUS) approved hospital authorization (AIH) values and do not include societal costs or inflation adjustments.

 Total hospital expenditures for AD-related admissions between 2018 and 2024 amounted to R$ 14,103,778.26. The average cost per admission peaked in 2019 (R$ 1,552.65), followed by high values in 2020 (R$ 1,396.97), and reached a minimum in 2021 (R$ 1,180.12). The highest total expenditure was recorded in 2023 (due to volume), while cost per case decreased slightly (R$ 1,195.27). No statistically significant trend was found for average hospitalization cost over time (p=0.1606) (Figure 4). 

### Costs by region

**Figure 5:**
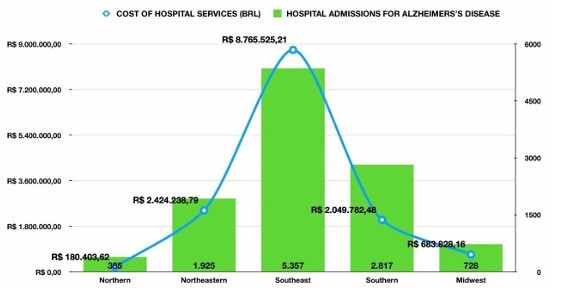
Hospitalization costs for Alzheimer’s disease by region, Brazil, 2018–2024. Bars indicate total regional expenditure in BRL; markers show the average cost per admission (BRL) within each region. Values are nominal BRL (no inflation adjustment). Notes: Costs reflect approved Hospital Information System of the Brazilian Unified Health System (SIH/SUS) approved hospital authorization (AIH) values and do not include societal costs or inflation adjustments.

 The Southeast region accounted for R$ 8,765,525.21 (62.2%) of the total cost, with the highest average cost per case (R$ 1,636.92). The other regions reported the following costs: South—R$ 2,049,782.48 (R$ 727.93), Northeast—R$ 2,424,238.79 (R$ 1,259.90), Midwest —R$ 683,828.16 (R$ 939.64), and North—R$ 180,403.62 (R$ 468.06) (Figure 5). 

## DISCUSSION

 This study provides an updated epidemiological overview of hospitalizations for AD in Brazil from 2018 to 2024, using data from the Hospital Information System of the Unified Health System (SIH/SUS)^18^. The period was selected to encompass pre-pandemic (2018 and 2019), pandemic (2020 and 2021), and post-pandemic (2022, 2023 and 2024) years, enabling analysis of COVID-19’s impact on AD-related hospital morbidity. This approach addresses key gaps left by prior studies, expanding demographic, regional, and economic insights into the disease. 

 Our results mirror international patterns in which AD hospitalizations are concentrated among women aged ≥80 years, urgent admissions constitute a large share of episodes, and resource use clusters in older age strata. Many countries documented a downturn in AD admissions in 2020 during the first pandemic wave, followed by a rebound in 2021–2023, consistent with our Brazilian time series. Cross-regional differences in hospitalization rates, length of stay, and costs observed here also parallel reports linking variability to demographic structure, multimorbidity and case-mix, care pathways, and access to specialized dementia services and post-acute care. Nevertheless, direct comparisons remain constrained by heterogeneity in data sources (administrative vs. clinical), coding practices, and health-system organization. By reporting nationwide crude and age-standardized rates with regression-based trends, our study provides metrics that are comparable to those in the international literature while reflecting Brazil’s specific service configuration and population aging profile^4,9,10^. 

 These points situate Brazil within global trends and support the external validity of our findings without overstating cross-country comparability. 

 The concentration of urgent admissions and the longer length of stay observed in our series (Table 1), together with rising utilization after the pandemic trough (Figure 1), indicate opportunities for system-level interventions to prevent potentially avoidable hospitalizations and contain costs. Priority actions include scaling memory clinics within primary care; proactive case management and caregiver support (education, respite, and navigation); standardized pathways for behavioral and psychological symptoms of dementia to reduce urgent referrals; early identification of frailty and common triggers of decompensation (infections, dehydration, medication-related events); and transitional care bundles at discharge (medication reconciliation, follow-up calls, and timely outpatient appointments). Regions with higher utilization and longer stays may benefit from targeted capacity building (community dementia services, post-acute and home-based care), while hospital protocols should incorporate delirium prevention, non-pharmacological management, and early goals-of-care discussions. From a financing perspective, routine monitoring of urgent admission rates, length of stay, re-hospitalization within 30 days, and in-hospital mortality for AD can inform performance-based commissioning and guide resource allocation. Given that SIH/SUS costs reflect Hospital Admission Authorization (AIH) values rather than full societal costs, complementary economic evaluations are warranted to estimate the budget impact and cost-effectiveness of the above strategies in the Brazilian context. 

 While descriptive data indicated an increase in absolute admissions in 2023, linear regression analysis found no statistically significant trend over the period (p=0.1558). This suggests the 2023 spike may reflect a transient fluctuation rather than a sustained pattern. These results contrast with Justo et al.^20^, who reported a decrease in dementia-related admissions, but align with findings from Feter et al.^21^ and Piovesan et al.^22^, who observed increases in AD admissions and associated costs between 2010 and 2019/2020, especially among women, individuals aged 80 or older, and residents of the Northeast and Midwest regions. 

 Urgent hospitalizations predominated (≈80%) and accounted for a higher share of in-hospital deaths (79.1%), reflecting the advanced stage of disease at admission. International studies, such as Sampson et al.^23^, suggest that up to half of the older adults admitted to urgent departments may have undiagnosed dementia. This may also occur in Brazil, potentially contributing to underdiagnosis and underreporting of AD. 

 Regional disparities were marked. The Southeast accounted for nearly half of all admissions (47.8%) and 62.2% of total hospital costs, while the North represented only 3.4% of cases and 2.3% of deaths. These differences may reflect not only population distribution and concentration of specialized services but also structural inequalities in access and diagnosis. Nitrini et al.^24^ highlight that, in middle-income countries such as Brazil, high-quality data and access to specialized dementia care remain limited. 

 Racial disparities also deserve attention. White and Pardo individuals comprised most hospitalizations, while Black Brazilians accounted for only 4.5% of cases and 6.5% of deaths. This may partly stem from lower life expectancy among Black Brazilians, as demonstrated by Chiavegatto Filho et al.^25^, reducing the likelihood of developing and being diagnosed with AD. Moreover, Suemoto et al.^26^ report that vascular dementia is more prevalent among Black patients, possibly contributing to underrepresentation in AD-specific registries. 

 The COVID-19 pandemic directly impacted hospitalization patterns. In 2020, AD-related hospitalizations were at their lowest, while hospital mortality was highest (26.9%) and average cost per admission peaked (R$ 1,396.97). This paradox may be explained by delayed care, greater clinical severity at admission, and increased spending on supplies and protective measures. Similar findings were reported by Carvalho et al.^27^ in the context of general hospitalizations during the pandemic. 

 Hospital cost analysis showed that the Southeast incurred the highest absolute and average expenditures, while the North reported the lowest, reflecting disparities in hospital infrastructure and access. Because cost data were not adjusted for inflation, longitudinal comparisons should be interpreted with caution (see Limitations). Nevertheless, no significant trend was observed in average hospitalization cost over time (p=0.1606), differing from global patterns of rising dementia-related healthcare spending. 

 This study relies on the SIH/SUS administrative database, which entails several sources of bias. First, diagnostic accuracy depends on correct ICD-10 coding of Alzheimer’s disease (G30*), making the data vulnerable to miscoding and underreporting, with potential heterogeneity across regions and over time. Second, the system does not distinguish incident admissions from readmissions, which may overestimate the number of unique patients. Third, SIH/SUS does not capture hospitalizations financed outside the public system; therefore, national estimates reflect the SUS-insured population and may not represent care in the private sector. Fourth, important clinical variables—such as comorbidity profiles, functional status, dementia severity, medication use, and complications—are not systematically available, limiting risk adjustment and case-mix comparisons. Fifth, events managed exclusively in outpatient or urgent settings without admission are not recorded, which can underestimate the overall healthcare burden. Sixth, hospital costs reflect AIH values and may not include all resource categories or indirect societal costs. Finally, in-hospital mortality should not be interpreted as disease-specific case fatality, because linkages to death registries and post-discharge outcomes were not feasible. These limitations notwithstanding, our age-standardized analyses and regression-based trends help mitigate confounding by demographic structure and support the internal consistency of the main findings. 

 In conclusion, AD places an increasing burden on Brazil’s public healthcare system, characterized by predominantly urgent hospitalizations, high in-hospital mortality, and significant costs. Observed regional and racial disparities should inform public health planning, with emphasis on early diagnosis strategies, decentralized care, and improved data quality. Alignment with international guidelines, such as those proposed by the Lancet Commission, is essential to ensure a structured national response to demographic aging in Brazil^28^. 

## Data Availability

All data supporting the findings of this study are publicly available through the open-access platform DATASUS (Departamento de Informática do Sistema Único de Saúde), accessible via https://datasus.saude.gov.br/. No individual-level or identifiable data were used, and therefore ethical approval was waived.
